# Pharmacovigilance Data as a Trigger to Identify Antimicrobial Resistance and Inappropriate Use of Antibiotics: A Study Using Reports from The Netherlands Pharmacovigilance Centre

**DOI:** 10.3390/antibiotics10121512

**Published:** 2021-12-09

**Authors:** Jean Marie Vianney Habarugira, Linda Härmark, Albert Figueras

**Affiliations:** 1 European & Developing Countries Clinical Trials Partnership (EDCTP), 2593 HW The Hague, The Netherlands; 2 Departament de Farmacologia, de Terapèutica i de Toxicologia, Universitat Autònoma de Barcelona (UAB), 08193 Barcelona, Spain; albert.figueras@gmail.com; 3 Netherlands Pharmacovigilance Centre Lareb, 5237 MH ′s-Hertogenbosch, The Netherlands; l.harmark@lareb.nl

**Keywords:** antibiotics, antimicrobial resistance, antimicrobial stewardship, AWaRe, pharmacovigilance, Lareb, adverse drug reactions

## Abstract

(1) Background: Antimicrobial resistance (AMR) requires urgent multidisciplinary solutions, and pharmacovigilance has the potential to strengthen current antimicrobial stewardship strategies. This study aimed to characterize AMR-relevant adverse drug reaction (ADR) reports submitted to The Netherlands Pharmacovigilance Centre; (2) Methods: We carried out a descriptive analysis of ADR reports submitted to Lareb, coded with AMR-relevant MedDRA Preferred Terms (PTs); (3) Results: Between 1998 and January 2019, 252 AMR-relevant ADR reports were submitted to Lareb. The most frequent antibiotics were tobramycin (*n* = 89; 35%), colistin (*n* = 30; 11.9%), cipro-floxacin (*n* = 16; 6.3%), doxycycline (*n* = 14; 5.5%), and aztreonam (*n* = 12; 4.8%). The PTs used included off label use (*n* = 91; 36.1%), drug ineffective (*n* = 71; 28.2%), product use in unapproved indication (*n* = 28; 11.1%), pathogen resistance (*n* = 14; 5.6%), and drug resistance (*n* = 13; 5.2%). 54% of the reports were on Watch antibiotics and 19% were involved in the Reserve group. In the Watch group, “off label use” and “product use in unapproved indication” were the most frequent PTs and the majority of reports on Reserve antibiotics were coded as “Off label”. A sharp increase in the number of reports was observed in the three consecutive years with 21 in 2013, 54 in 2014, and 83 in 2015; (4) Conclusions: In addition to existing AMR monitoring strategies, pharmacovigilance databases can serve as a source of data on suspected resistance and inappropriate use. Future research should explore how these AMR-relevant MedDRA Terms are used in resource-limited settings with less capacity to generate laboratory-confirmed resistance data.

## 1. Introduction

### 1.1. Antimicrobial Resistance (AMR) and Antimicrobial Stewardship

Antimicrobials include a broad range of medicines used to prevent and treat infections in humans, animals, and plants [1]. These medicines are designed to kill or inhibit the growth of microorganisms responsible for infections. However, with time antimicrobial resistance (AMR) occurs as the same microorganisms develop the ability to resist the antimicrobial action of previously effective medicines [2]. Antibiotic resistance affects people of all ages in all countries. Yearly, an estimated 5.7 million deaths occur from treatable infectious diseases, mostly in low- and middle-income countries (LMICs), and many of these lives could have been saved if effective antibiotics were available. At the same time, there are about 700,000 annual deaths worldwide due to antibiotic resistance. The development of resistance, while threatening the right to the best medical care, has also shown us that resource-limited settings are more affected by lack of access to antibiotics than by resistance.

Antimicrobial stewardship refers to efforts that encourage the responsible use of antimicrobials with the aim of minimizing the development of resistance and including actions that minimize dose and duration of treatment and ensure use of the correct antibiotic [3,4]. Whilst measures have been put in place to address AMR at hospital or country levels, there is still lack of a globally coordinated strategy to curb increasing resistance [5]. A global coordination is challenged by limited antimicrobial stewardship interventions at the level of primary health care, the dispensing of antimicrobials without prescription in pharmacies, and the extended use of antimicrobials in non-human sectors [6]. As a program, antimicrobial stewardship is a set of all interventions used to enhance the rational use of antibiotics [7]. Various forms of antimicrobial stewardship programs have been established in different countries [8] at different levels of care delivery with involvement of a wide range of stakeholders including clinicians, pharmacists, nurses, and administrators and healthcare facilities [9,10,11].

Preserving antibiotic effectiveness while ensuring universal access is at the heart of public health dilemmas, as policies for good access must be accompanied with strong measures to minimize inappropriate use that would lead to further resistance [12,13].

### 1.2. The Access, Watch and Reserve (AWaRe) Classification for Availability and Appropriate Use

To address the issue of availability while ensuring appropriate use, since 2017, the Word Health Organization (WHO) Essential Medicines List (EML) includes a classification of antimicrobials into three categories known as “Access”, “Watch”, and “Reserve” (AWaRe), based on the indication, availability, and awareness [14]. A global campaign was launched in 2019 urging governments to implement the AWaRe tool through national guidelines to reduce antimicrobial resistance and ensure access [15].

As the world puts in place various measures to curb the rising threat caused by the rising resistance to existing antibiotics, specific pharmacovigilance data could constitute an important part of the wider multi-disciplinary approaches used for resistance surveillance and warning. The AWaRe classification is a useful tool to be considered by monitoring activities targeting specific antibiotics.

### 1.3. Pharmacovigilance and Antimicrobial Resistance

Pharmacovigilance is the science of the activities relating to the detection, assessment, understanding and prevention of adverse effects or any other possible drug-related problems [16]. Adverse effects and other drug-related problems include a range of negative or harmful patient outcomes that seem to be associated with treatment. An adverse drug reaction (ADR) is referred to when causality assessment has taken place and the link between the medicine and a suspected adverse effect is beyond uncertainty [17]. In the context of this study, the term adverse drug reaction (ADR) report refers to reports sent by health professionals or patients when an adverse effect has occurred in a patient taking one or more antimicrobial. The scientific community continues to propose innovative antimicrobials monitoring approaches, and some have suggested pharmacovigilance data as a potential source of information for antimicrobial stewardship programs [18]. Recent studies have underlined the potential role of Pharmacovigilance in containing rising antimicrobial resistance [19,20], proposing methods and tools that pharmacovigilance can offer to programs that monitor suspected resistance or cases of inappropriate use of antimicrobials. A study conducted in Russia concluded that the most frequent types of medication errors (“administration of an antibiotic in the absence of indications/wrong indication”, “incorrect dosage and regimen”, and “administration of a contraindicated drug”) associated with the use of beta-lactams were the leading risk factors of growing bacterial resistance [21]. A timely reporting and correct coding of such errors in a spontaneous reporting database can inform policy on appropriate use of antimicrobials. Other researchers have looked at therapeutic failure as a reportable event but recognized the need to use the right definition of failure [22] if pharmacovigilance systems are to be systematically used for collection data on failure. Other studies have emphasized the importance of pharmacovigilance databases which constitute a unique resource of information on potential misuse of medicines (including antimicrobials) and information potentially containing AMR-relevant data [23]. At least one study has identified a set of 17 MedDRA preferred terms (PTs) which can be used to generate data related to concepts such as resistance, ineffectiveness, off-label use, and medication errors (RIOLE) [24]. In a recent study conducted in India, the authors agree that ADR reports associated with antibiotics can facilitate the development of policies for appropriate use of antibiotics, thereby contributing to the fight against antimicrobial resistance [25].

### 1.4. Pharmacovigilance in The Netherlands

Established in 1968, the WHO Program for International Drug Monitoring (PIDM) had The Netherlands as one of its ten founding members. Since then, the program has expanded to include 145 full member countries and 26 associate member countries [26]. The Netherlands Pharmacovigilance Center Lareb manages a spontaneous reporting system, which involves collection and evaluation of suspected adverse drug reactions (ADRs) of medicinal products aiming to identify new safety signals. Lareb codes the reports using MedDRA, and the reports are assessed by qualified assessors before entry into the database and prior to sharing with the European and global community via the Eudravigilance database of the European Medicines Agency (EMA) and VigiBase, the database of the PIDM. To determine if a specific drug-reaction combination reported could constitute a signal, a scientific review must take place. Identified safety signals are shared with the Dutch Medicines Evaluation Board (CBG/MEB) which will decide, often in the European context, if further regulatory action is necessary [27]. As a founding member of the PIDM, The Netherlands has a rich experience in ADR reports collection and sharing with the world through the European and global database. One strength of the Dutch system is that it collects reports directly via the patient reporting scheme initiated in 2003, which is now considered as a reliable of source of safety data [28,29].

### 1.5. Study Objective

The objective of this study was to characterize ADR reports in the database of The Netherlands Pharmacovigilance Centre (Lareb) following use of antibiotics and coded with MedDRA Preferred Terms that suggest suspicion of resistance, ineffectiveness, off-label use, or medication errors (RIOLE).

## 2. Material and Methods

### Data Source and Search Strategy

We carried out a descriptive analysis of ADR reports in the database of The Netherlands Pharmacovigilance Center (Lareb) database. Patients and healthcare professionals can report directly to Lareb. Marketing authorization holders reports directly to Eudravigilance. The reports are then forwarded to Lareb so that Lareb has a complete overview of reported ADRs which have occurred in the Netherlands. Reports fulfilling the following criteria were included:

(a) Reports on Antibiotics classified under the Anatomical Therapeutic Chemical (ATC) Classification ATC J01 or ATC J04.
(b) Reports coded with at least one of the following MedDRA (version 21.1) Preferred terms and codes included the following: pathogen resistance (10034133); drug ineffective (10013709); treatment failure (10066901); drug resistance (10059866); therapeutic product ineffective (10060769); therapy non-responder (10051082); decreased activity (10011953); drug ineffective for unapproved indication (10051118); therapeutic response decreased (10043414); multiple drug resistance (10048723); off label use (10053762); medication error (10027091); product use in unapproved indication (10076476); contraindicated product administered (10078504)

For each report meeting the criteria in (a, b), the following information was collected and included in the dataset for further analysis: report identifier; suspected ADR; year of report; reporter type; suspected drug; indication and action taken.

## 3. Results

### 3.1. ADR Reports with AMR-Relevant Codes

Between 1998 and January 2019, a total of 252 ADR reports (study sample) were submitted to Lareb using a PT or a combination of PTs that suggested suspicion of AMR or use-related issues (irrational use or medication errors). The following antibiotics were the most frequently reported as suspected causes of AMR-relevant ADRs: tobramycin (*n* = 89; 35%), colistin (*n* = 30; 11.9%), ciprofloxacin (*n* = 16; 6.3%), doxycycline (*n* = 14; 5.5%), and aztreonam (*n* = 12; 4.8%). The most frequently used PTs were off label use (*n* = 91; 36.1%), drug ineffective (*n* = 71; 28.2%), product use in unapproved indication (*n* = 28; 11.1%), pathogen resistance (*n* = 14; 5.6%), and drug resistance (*n* = 13; 5.2%).

### 3.2. Most Frequently Used PTs in Cases of Suspected Resistance or Use-Related Issues

As shown in Table 1, 98 reports (39% of the study sample) suggested suspicion of resistance using PTs such as drug ineffective, pathogen resistance, and drug resistance. 119 reports (47% of the study sample) included PTs suggesting use-related issues such as off label use (*n* = 91; 76%) and product use in unapproved indication (*n* = 28; 15%). More than half of the 91 reports coded with PT off label use described events in patients on tobramycin as the suspect drug (*n* = 53; 58%); additionally, 24 (26%) were on colistin. The reports coded as product use in unapproved indication were predominantly on tobramycin (*n* = 27; 96% of the cases). Out of the 252 reports of the study sample, 35 (14%) were coded each with more than 1 PT, combining PTs that refer to suspicion of both resistance and use-related issues.

### 3.3. Applying the AWaRe Classification to the Reports

As shown in Table 2, the Watch category was involved in 137 (54%) of the 252 ADR reports with a predominance of tobramycin with 89 of the 147 Watch reports (78%). The second leading group is Reserve with 45 reports (19%), followed by the Access group with 40 reports (16%), and the remaining 11% include combination of antibiotics from different AWaRe groups or non-AWaRe classified antibiotics. In the Watch group, “off label use” and “product use in unapproved indication” were the most frequent PTs, used in 57 (42%) and 27 (20%) reports, respectively. The majority (76%) of reports involving Reserve antibiotics were submitted as off label use. In the Access group, “drug ineffective” was the most frequent PT in 25 (63%) out of 40 reports.

The 91 off label use ADR reports include predominantly tobramycin (Watch) and colistin (Reserve) as the suspected drug with 53 (58%) and 24 (26%) reports, respectively.

### 3.4. The 2015 Peak in Numbers of AMR-Relevant ADR Reports to Lareb

**Figure 1:**
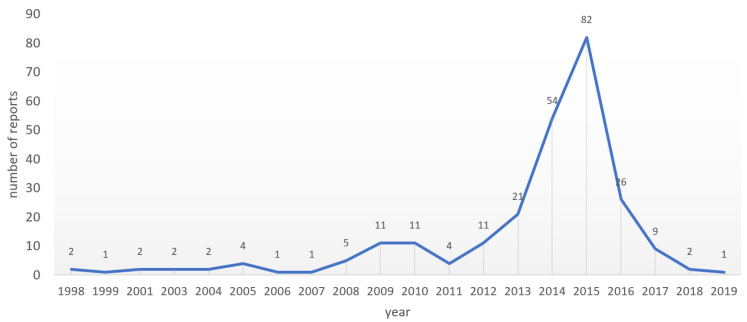
Yearly reports from 1998 to Jan 2019.

From the 252 ADR reports submitted over a period of about 20 years, 82 (34%) reports were submitted in 2015 as illustrated by Figure 1. A sharp increase in the number of reports was observed in the three consecutive years with 21 in 2013, 54 in 2014, and 83 in 2015. The numbers dropped to 26 reports in 2016, but in just these four years, Lareb received 73% (183 of 252) of AMR-relevant reports received over a period of 20 years. In this short period of sharp increase, there was a clear increase of tobramycin reports passing from 11 in 2013 to 30 in 2014 and reaching 39 in 2015. Colistin shows a visible increase of ADR reports in this period, passing from 0 in 2013 to 10 in 2014 and 14 in 2015. Other drugs with increasing ADR reports numbers in the spike period include aztreonam and doxycycline. Of the 183 reports submitted in the period 2013–2016, 84 reports were on suspected “off label use” with a predominance of tobramycin that was the suspect drug in 50 (of 84) reports. 39 of the 183 reports were submitted with the PT “drug ineffective” and diverse antibiotics were reported as suspected drugs. However, with reports coded using the PT “product use in unapproved indication” indication, a clear predominance of tobramycin was observed.

## 4. Discussion and Conclusions

As the world seeks and puts in place strategies to tackle rising AMR, living reviews of pharmacovigilance data should be seen as a strong potential source of data on trends in suspected resistance and possible irrational use of antimicrobials. The present analysis of the Lareb database has shown that the AMR-relevant preferred terms can be identified in a national spontaneous reporting database.

These pharmacovigilance data can signal use-related issues which can be used to provide a bigger picture to prescribers who have a choice to make during each consultation. Reports on off-label use or drug use in contraindicated indications can be shared with entities responsible for national policies on antimicrobials use to timely inform the decision-making process. The pharmacovigilance data also identified reports with antibiotics belonging to the Reserve and Watch list, which is of special interest from an AMR perspective. Analysis of these reports can possibly lead to information regarding how and why different Watch and Reserve antibiotics are potentially overused or used off-label, depending on the indications and available formulations. In this study we have noted an increased number of reports in the period from 2013 to 2015. A spike in reports can also signal that something has changed regarding the drug. By analyzing a reporting spike, it is possible to see if there are changing in how a drug is being used.

In this study, most reports were submitted by manufacturers, and this makes it more difficult to interpret what the data mean in the context of identifying AMR-related issues. It should be noted that The Netherlands has an advanced regulatory pharmacovigilance system with stringent rules for reporting (especially for MAHs). The surge in reports needs to be interpreted in the light of both clinical relevance as well as reporting obligations for MAHs. It is not clear if the spike represents a difference in use or just that it was not reported in earlier years. The 2015 peak may be related to an intensified reporting from specific MAHs responding to reporting obligations, following up specific cohorts involving for example tobramycin and colistin. However, without more detailed reports from the responsible MAH, it is impossible to have a solid explanation of the reasons behind the peak. Therefore, future research focusing of this intersection between antimicrobial resistance and pharmacovigilance should also pay more attention to the specific reporting obligations that MAHs must comply with.

In many countries, health care professionals play a crucial role in gathering and submitting safety data to competent authorities. Manufacturers rely on health care professionals working on clinical trials or seeing patients in clinics to provide solicited or unsolicited safety data on products. According to the Guideline on Good Pharmacovigilance Practices Module V, in their risk management plans, manufacturers are required to address undesirable clinical outcomes for which there is sufficient scientific evidence that they are caused by the medicinal product [30].

If the drug safety community agrees that resistance or ineffectiveness are undesirable clinical outcomes following use of antimicrobials with the intention to prevent or treat a disease, the manufacturers should consider the use of AMR-relevant MedDRA terms in collecting data to inform their risk management plans. The Guideline on Good Pharmacovigilance Practices also indicates that reports of adverse reactions may be derived from multiple sources including spontaneous data sources and may be linked to situations such as off label use and medication errors. If the concerned medicinal product is antimicrobial, competent authorities should consider the risk associated with off label use and medication in the antimicrobial stewardship context.

This study shows that a national pharmacovigilance database has reports coded with AMR-relevant PTs. However, as some pharmacovigilance centers may have limited capacity and skills to assess received ADR reports and assign them with a correct code, the replicability of this study could be difficult, especially in countries with a weak pharmacovigilance system.

Further, as this study was conducted in a country with stringent regulations for manufacturers, the reproducibility could be difficult in countries with weak regulatory capacity.

Expanding the role of pharmacovigilance requires both promotion of existing tools and education for the potential reporters of observed or suspected effects. In this study, the analyzed reports include those submitted by manufacturers, and there may be other potential similar cases that went unreported by prescribers and patients.

Promoting the existence of AMR-relevant MedDRA terms and explaining their relevance to surveillance of antimicrobial resistance could lead to increased submission of similar reports from prescribers and patients. Public health will gain from different angles if pharmacovigilance is integrated in the antimicrobial stewardship programs package. pharmacovigilance centers at country level should be encouraged to actively promote these AMR relevant PTs and invite reporters to collect and send this information to relevant agencies. By getting actively involved in this process: (1) pharmacovigilance centers will receive more spontaneous reports, and the centers’ work will be more visible and appreciated by other public health stakeholders; and (2) suspected AMR could be timely detected, potential clusters could be highlighted and, if necessary, ad-hoc microbiological tests could be conducted. The integration of pharmacovigilance in this process could lead to a win-win situation for different scientific disciplines tackling AMR from traditionally isolated perspectives. Pharmacovigilance is already a multidimensional science with potential to expand further. Tackling AMR questions using the existing pharmacovigilance methods will also provide an opportunity for pharmacovigilance as a field to evolve further and countries will see more value in investing in their pharmacovigilance systems. As technology further advances the field of pharmacovigilance, methods traditionally used in post-marketing drug safety monitoring have the potential to serve as surveillance strategies for antimicrobial stewardship programs.

## Figures and Tables

**Table 1:** The most frequently used PTs and reported antibiotics per RIOLE group.

RIOLE Categories (Number of Reports; %)	PTs in the RIOLE Category (Number of Reports; %)	Most Reported Antibiotics per PT (Number of Reports; %)
Suggesting AMR (98; 39%)	drug ineffective (71; 72%),	aztreonam (9; 13%)
		amoxicillin + Beta-lactamase inhibitor (6; 8%)
		doxycycline (6; 8%)
	pathogen resistance (14; 14%)	ceftazidime (5; 36%)
		ciprofloxacin (2; 14%)
		linezolid (2; 14%)
	drug resistance (13; 13%)	tobramycin (3; 23%)
		ciprofloxacin (2; 15%)
Suggesting use-related issues (119; 47%)	off-label use (91; 76%)	tobramycin (53; 58%)
		colistin (24; 26%)
		doxycycline (6; 7%)
	product use in unapproved indication (28; 15%)	tobramycin (27; 96%)
Suggesting both AMR and use-related issues (35; 14%)	Combinations of PTs Suggesting both AMR and use-related issues (35; 14%)	ciprofloxacin (7; 20%) azithromycin (3; 9%)
TOTAL = 252; 100%	-	-

**Table 2:** The most reported antibiotics and used PTs per AWaRe class.

AWaRe Categories (Number of Reports; %)	Most Reported Antibiotics in the AWaRe Category (Number of Reports; %)	Most Used PTs in the AWaRE Category (Number of Reports; %) *
Access (40; 16%)	doxycycline (14; 35%)	drug ineffective (25; 63%) off label use (6; 15%)
	amoxicillin + Beta-lactamase inhibitor (7; 18%)	
	sulfamethoxazole + trimethoprim (4; 10%)	
Watch (137; 54%)	tobramycin (89; 78%)	Off label use (57; 42%) Product use in unapproved indication (27; 20%) drug ineffective (20; 15%) pathogen resistance (8; 6%)
	ciprofloxacin (16; 33%)	
	azithromycin (8; 17%)	
	moxifloxacin (7; 15%)	
Reserve (45; 19%)	colistin (30; 91%)	off label use (25; 76%)
	aztreonam (12; 11%)	
Combination of different classes (17; 6%)	concomitant from different classes	drug ineffective (7; 41%) drug resistance (3; 18%)
Other or not classified (13; 5%)		drug ineffective (8; 61%)
TOTAL (252; 100%)	-	-

* Most frequently used PT per AWaRe class (Access, Watch, Reserve).

## Data Availability

Restrictions apply to the availability of these data. Data was obtained from Lareb and are available from the authors with the permission of Lareb.
